# Parental diagnostic delay and developmental outcomes in congenital and childhood‐onset myotonic dystrophy type 1

**DOI:** 10.1111/dmcn.16079

**Published:** 2024-09-04

**Authors:** Federica Trucco, Emilio Albamonte, Marika Pane, Federica Ricci, Adele D'amico, Guja Astrea, Isabella Moroni, Antonella Pini, Chiara Fiorillo, Angela Berardinelli, Nicholas E. Johnson, Valeria A. Sansone, The DM1 Paediatric Working Group, Andrea Lizio, Alessandra di Bari, Francesca Salmin, Maria Beretta, Jacopo Casiraghi, Susanna Pozzi, Laura Antonaci, Anna Salvalaggio, Michela Catteruccia, Michele Tosi, Gemma Marinella, Federica R. Danti, Fabio Bruschi, Gaia Scarpini, Marco Veneruso, Stefano Parravicini, Amanda Ferrero, Barbara Risi, Andrea Barp, Riccardo Zuccarino, Massimilano Filosto, Michela Coccia, Roberta Battini, Eugenio Mercuri, Kiera Berggren

**Affiliations:** ^1^ NeMO Clinical Center, Fondazione Serena Milan Italy; ^2^ Department of Neurorehabilitation University of Milan Milan Italy; ^3^ Paediatric Neurology and Muscular Diseases Unit, Istituto di Ricovero e Cura a Carattere Scientifico Istituto Giannina Gaslini, Department of Neurosciences, Rehabilitation, Ophthalmology, Genetics, Maternal and Child Health University of Genova Genoa Italy; ^4^ Centro Clinico NeMO Fondazione Policlinico Universitario Agostino Gemelli Istituto di Ricovero e Cura a Carattere Scientifico Rome Italy; ^5^ Paediatric Neurology Università Cattolica del Sacro Cuore Rome Italy; ^6^ Department of Sciences of Public Health and Pediatrics University of Turin Turin Italy; ^7^ Unit of Muscular and Neurodegenerative Disorders, Bambino Gesù Children's Hospital Istituto di Ricovero e Cura a Carattere Scientifico Rome Italy; ^8^ Department of Developmental Neuroscience Istituto di Ricovero e Cura a Carattere Scientifico Fondazione Stella Maris Pisa Italy; ^9^ Department of Pediatric Neurosciences Fondazione Istituto di Ricovero e Cura a Carattere Scientifico Istituto Neurologico Carlo Besta Milan Italy; ^10^ Pediatric Neuromuscular Unit, UOC Neuropsichiatria Dell'Età Pediatrica Istituto di Ricovero e Cura a Carattere Scientifico Istituto delle Scienze Neurologiche di Bologna Bologna Italy; ^11^ Unit of Child Neuropsychiatry Istituto di Ricovero e Cura a Carattere Scientifico Istituto Giannina Gaslini and DINOGMI, University of Genova Genoa Italy; ^12^ Child and Adolescent Neuromuscular Disorder Unit Istituto di Ricovero e Cura a Carattere Scientifico Mondino Foundation Pavia Italy; ^13^ Department of Neurology Virginia Commonwealth University Richmond VA USA

## Abstract

**Aim:**

To investigate the timing of type 1 myotonic dystrophy (DM1) diagnosis in parents of affected children and describe children's perinatal characteristics and developmental outcomes.

**Method:**

This was a descriptive case series of children with congenital myotonic dystrophy (CDM) and childhood‐onset myotonic dystrophy (ChDM). Parental timing of DM1 diagnosis and the perinatal, motor, and cognitive outcomes of paediatric patients were recorded.

**Results:**

A total of 139 children followed by 12 highly specialized tertiary care neuromuscular centres in Italy and one tertiary neuromuscular centre in the USA were included: 105 children with CDM and 34 children with ChDM (mean age 8 years 8 months and 12 years 2 months respectively; 49 males and 17 males respectively). Seventy (50%) parents were diagnosed with adult‐onset DM1 after the affected child was diagnosed. Only 12 (17%) of the 69 parents known to be affected had prenatal testing.

Of the 105 children with CDM, 98% had maternally inherited CDM, 36% were born preterm, 83% required a stay in the neonatal intensive care unit for more than 48 hours, 84% and 79% had ambulation and speech delay, and 84% had an IQ lower than 70. Of the 34 children with ChDM, 59% had paternally inherited ChDM, 91% were born at term, and 36% had an IQ lower than 70.

**Interpretation:**

Delay in diagnosing DM1 affects family planning. The prenatal and perinatal outcomes of the affected offspring emphasize the need for proactive counselling as parents may be reluctant to conduct prenatal testing.

## Abstract 2

Plain language summary: https://onlinelibrary.wiley.com/doi/10.1111/dmcn.16150

This original article is commented by Nakamura on pages 281–282 of this issue.

**Abbreviations**
CDM: congenital myotonic dystrophy
ChDM: childhood‐onset myotonic dystrophy
DM1: type 1 myotonic dystrophy
NICU: neonatal intensive care unit

**What this paper adds**

- A parental diagnosis of type 1 myotonic dystrophy (DM1) was known in only 50% of pregnancies in this cohort.
- A minority of affected individuals (17%) had prenatal testing before conception or during pregnancy.
- A delay in diagnosing DM1 impacts family planning and highlights the need for proactive prenatal counselling.
- Disease burden in congenital and childhood DM1 is high.
- Early parental diagnosis is required to plan delivery and for timely management of the emerging complications.

Type 1 myotonic dystrophy (DM1) is an autosomal dominant disorder caused by a CTG trinucleotide repeat expansion in the *DMPK* gene leading to the aberrant splicing of several effector genes via the nuclear sequestration of RNA‐binding proteins.^1^, ^2^ DM1 is the most common muscular dystrophy of adulthood, with an estimated prevalence of 1 per 2500 when considering individuals with a CTG trinucleotide repeat expansion of 50 or greater at birth.^3^ Anticipation is a feature of the disease. The expansion of the CTG trinucleotide repeat between generations results in the offspring of individuals with DM1 having a more severe phenotype, with onset either at birth (congenital myotonic dystrophy [CDM]) or during childhood (childhood‐onset myotonic dystrophy [ChDM]).^4^

Muscle impairment in adult‐onset DM1 usually presents in late adolescence or young adulthood and is characterized by grip myotonia, which represents the hallmark of the disease, and distal muscle weakness. These symptoms can often be deceiving and are not easily recognized, especially in adults who are mildly affected. Myotonia is quite variable in severity, is usually transient depending on temperature, and improves with repeated exercise. Patients often underestimate ‘locking’ or ‘stiffness’ in the hands and do not report them as part of their clinical history. Cardiac manifestations are also common in DM1. First‐degree atrioventricular blocks are frequent, but these are usually asymptomatic and are rarely the cause of medical attention in the initial phases of the disease.

DM1 is also considered a ‘brain disorder’.^5^ Central nervous system involvement^6^, ^7^, ^8^ may be associated with central fatigue and excessive daytime sleepiness or may be characterized by frontal dysexecutive symptoms, such as apathy and lack of insight.^9^, ^10^, ^11^ DM1 is characterized by a broad spectrum of presentations that cause a significant burden on the affected individuals.^12^

Multiorgan involvement, the lack of awareness that patients attribute to their symptoms, and the relatively low prevalence of this disease, make it hard to diagnose it in the early stages.^13^ While the lag between symptom onset and genetic confirmation is recognized as a common finding in adult patients with DM1,^13^ less is known about the impact that this diagnostic delay has on family planning and family burden when infants with the congenital form survive and grow into childhood or when children with childhood‐onset DM1 approach school and social interactions increase.

The aim of this study was twofold: first, to investigate the rate of couples of childbearing age who received their DM1 diagnosis before or after the birth of a child diagnosed with CDM or ChDM and second, to describe the perinatal characteristics and developmental outcomes of children with CDM and ChDM.

## METHOD

### Study design

This study was a descriptive case series of children with either congenital or childhood‐onset DM1, followed by 12 highly specialized tertiary care neuromuscular centres in Italy (NeMO Clinical Center, Milan; Istituto Neurologico Carlo Besta, Milan; Istituto Neurologico Casimiro Mondino, Pavia; Istituto Giannina Gaslini, Genoa; Istituto delle Scienze Neurologiche, Bologna; Fodazione Stella Maris, Pisa; Fondazione Policlinico Universitario Agostino Gemelli, Rome; Ospedale Pediatrico Bambino Gesù, Rome; Ospedale Regina Margherita, Turin; Centro Clinico NeMO, Ancona; Centro Clinico NeMO, Brescia; Centro Clinico NeMO, Trento), and one tertiary neuromuscular centre at the Virginia Commonwealth University in the USA. Information was collected at subsequent time points from 2010 until the latest visit available before June 2022.

This study was part of a broader natural history study in paediatric‐onset DM1 in Italy and the USA and Canada, with annual study visits. In Italy, it was approved at all sites by the local Ethics Committee and Institutional Review Board (Research Ethics Committee 525–102019). In the USA and Canada, the study only included children with CDM. Recruitment was conducted non‐selectively through the ClinicalTrials.gov website for participants enrolled from 2016 to the present (ClinicalTrials.gov registration: NCT03059264), and through advertising at local neuromuscular clinic visits. Data regarding clinical outcomes were collected in patients enrolled in the study; information regarding parental diagnosis at the time of birth of each patient was gathered from medical records.

As information on parental diagnosis and parental disease status could not be retrieved for patients enrolled at the Canadian site, this group was not included in the analysis. Written informed consent was obtained from all participants (or guardians of participants) in the study (consent for research).

### Participants

This study included paediatric patients (younger than 18 years) with genetic testing confirming a CTG trinucleotide repeat expansion in the *DMPK* gene.

The class of CTG trinucleotide repeat expansion was recorded for both child and parent affected. Based on the number of CTG trinucleotide repeats, the class of expansion was classified as E1 (200–500 CTG trinucleotide repeats), E2 (500–1000 CTG trinucleotide repeats), E3 (1000–1500 CTG trinucleotide repeats), and E4 (> 1500 CTG trinucleotide repeats).^14^

When symptom onset in children occurred at birth (specifically within 30 days from birth), this was defined as CDM. Symptom onset after the newborn period and up to 10 years of age was defined as ChDM.^15^

### Timing of parental diagnosis

Parental timing of diagnosis (i.e. for any affected parent) was recorded for all children included in the study. Information regarding maternal or paternal inheritance and the timing of parental diagnosis defined as either before or after childbirth was collected.

When the diagnosis of DM1 in either of the two parents was only made after conception because of the child being affected, this was classified as a delay in diagnosis. The reasons for the delay in diagnosis, if any, were also recorded.

If the diagnosis of DM1 in either parent was known before the birth of the child, age and the main symptoms leading to the diagnosis were collected. Parental choice of whether to conduct prenatal testing because of the known diagnosis was also investigated.

### Short‐term and long‐term developmental outcome measures

The perinatal features of all children included in the study, namely the presence of respiratory distress, hypotonia, talipes (clubfoot), and facial weakness were collected. The requirement for non‐invasive or invasive ventilatory support, and the need for a stay in the neonatal intensive care unit (NICU) and the length of the stay, were also recorded.

The timing of acquisition of motor and cognitive developmental milestones were collected and compared to the physiological milestones defined by the World Health Organization.^16^

Intellectual ability was assessed using standardized age‐appropriate measures, namely the Wechsler Preschool and Primary Scales of Intelligence, Third Edition for ages 2 years 6 months to 7 years 3 months, or the Wechsler Intelligence Scale for Children, Fourth Edition for ages 6 years to 16 years 11 months. An IQ below 70 was defined as an extremely low cognitive function as per the Diagnostic and Statistical Manual of Mental Disorders, Fourth Edition.

Finally, the long‐term outcomes on motor (the ability to walk for more than 10 metres), respiratory (the requirement of non‐invasive ventilation), and nutritional (the ability to eat orally) abilities were recorded at the latest available visit.

### Statistical analysis

The distribution of each of the outcome variables was assessed using the Shapiro–Wilk test to test for normality.

The descriptive analysis and the results of the primary and secondary aims were evaluated with the mean and SD. The median and interquartile range (IQR) were used for continuous variables (as appropriate according to the type of distribution, that is, whether normal or not), while frequency and percentage were used for categorical variables. Analyses were performed using SAS v9.3 (SAS Institute, Cary, NC, USA).

## RESULTS

### Study sample and inheritance

A total of 139 children were included in the study; 105 fulfilled the criteria for CDM. Their median (IQR) age at the latest recorded visit was 8 years 8 months (5 years 2 months to 13 years 2 months). Thirty‐four patients were classified as having ChDM; their median (IQR) age was 12 years 2 months (7 years 8 months to 16 years 4 months). Ninety‐eight per cent (103 of 105) of children with CDM maternally inherited the disease; 59% (20 of 34) of children with ChDM paternally inherited the disease. The main features of the study sample are summarized in Table 1.

**TABLE 1:** Study sample (*n* = 139).

Characteristic	CDM	ChDM
Subsample size	105 (76)	34 (24)
Sex		
Male	49 (47)	17 (50)
Study site		
Italy	48 (46)	34 (100)
USA	57 (54)	0 (0)
Class of CTG trinucleotide repeat expansion		
E1 (200–500)	2 (2)	14 (41)
E2 (500–1000)	36 (34)	16 (47)
E3 (1000–1500)	43 (41)	3 (9)
E4 (> 1500)	14 (13)	1 (3)
Unknown	10 (10)	0 (0)
Age at disease onset (years:months), median (IQR)	0 (0–0)	6:6 (3:0–11:2)

All data are presented as *n* (%) unless otherwise indicated. CTG trinucleotide repeat expansion was classified as E1 (200–500 CTG trinucleotide repeats), E2 (500–1000 CTG trinucleotide repeats), E3 (1000–1500 CTG trinucleotide repeats), and E4 (> 1500 CTG trinucleotide repeats).^14^ Abbreviations: CDM, congenital myotonic dystrophy; ChDM, childhood‐onset myotonic dystrophy; IQR, interquartile range.

### Inheritance and timing of parental diagnosis

The diagnosis of DM1 was known at the time of conception only in 69 of 139 (50%) couples. Information regarding age and symptoms at disease onset was available for 14 of these 69 affected parents (10 females and four males). The median (IQR) age at symptom onset was 26 years 6 months (22 years 6 months to 30 years 4 months) in females and 36 years 6 months (21 years to 56 years) in males. Most of these parents (*n* = 8) were aware of their diagnosis because of the presence of an affected family member. In the remaining six parents, the most frequent disease signs leading to the diagnosis were daytime sleepiness, hand myotonia and weakness, and leg pain.

Only a minority (12 of 69; 17%) of couples with an affected family member decided to carry out prenatal diagnostic testing to screen for DM1 in their offspring. Information on the timing of diagnosis and the choice to conduct prenatal testing was available for couples whose offspring had either CDM or ChDM.

Of the 105 couples with a child with CDM, 55 (52%) had been diagnosed before conception; a prenatal diagnostic test was carried out only in 4 of 55 cases (7%). In the remaining 50 couples, diagnosis of DM1 in the mother was made only when their newborn infant was diagnosed with CDM.

In children diagnosed with ChDM, 14 of 34 parents (41%) were aware of the diagnosis at conception; 8 of 14 parents (57%) had a prenatal diagnostic test of their offspring during pregnancy (Table 2).

**TABLE 2:** Timing of parental diagnosis.

Timing	Overall (*n* = 139)	CDM (*n* = 105)	ChDM (*n* = 34)
Known parental diagnosis at conception	69/139 (50)	55/105 (52)	14/34 (41)
Choice of whether to conduct prenatal testing	12/69 (17)	4/55 (7)	8/14 (57)
Parental diagnosis of DM1 made when the affected child was born	70/139 (50)	50/105 (48)	20/34 (59)

All data are presented as *n* (%). Abbreviations: CDM, congenital myotonic dystrophy; ChDM, childhood‐onset myotonic dystrophy; DM1, type 1 myotonic dystrophy.

### Perinatal complications, developmental milestones, and long‐term outcomes

Polyhydramnios and reduced fetal movements were the most frequent findings during pregnancy in children born with CDM, with both occurring in 51% of pregnancies. Twenty‐six per cent of children with CDM had ventriculomegaly diagnosed before birth. None of the children with ChDM had prenatal complications.

Thirty‐six per cent of patients with CDM and 9% with ChDM were born preterm, defined as before 37 weeks gestational age.

**FIGURE 1:**
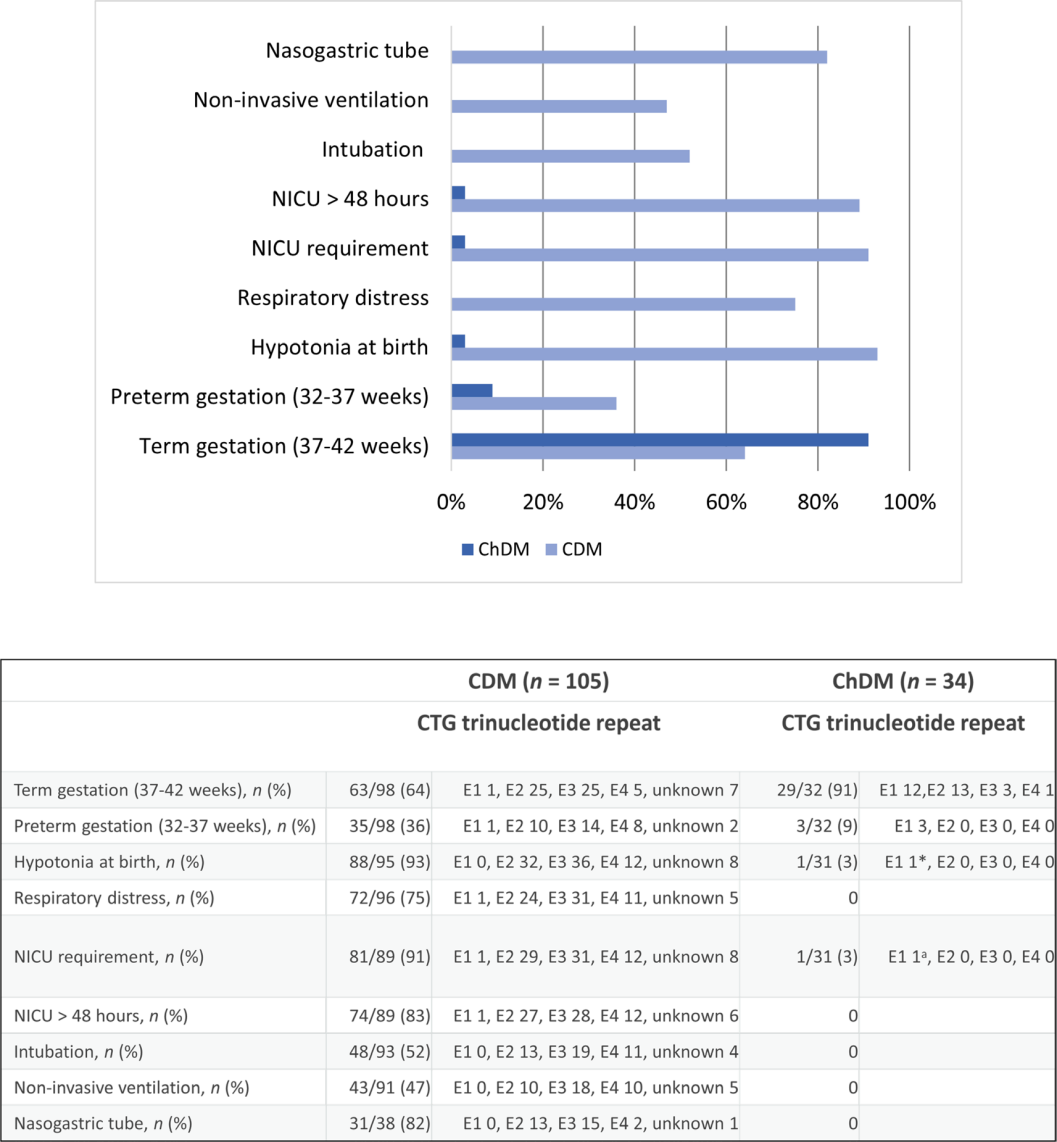
Perinatal complications in children with congenital myotonic dystrophy (CDM) and childhood‐onset myotonic dystrophy (ChDM). The classes of CTG expansion are: E1 (200–500 CTG trinucleotide repeats); E2 (500–1000 CTG trinucleotide repeats); E3 (1000–1500 CTG trinucleotide repeats); and E4 (> 1500 CTG trinucleotide repeats).^14^ ^a^One patient with a CTG expansion corresponding to E1 had mild neonatal hypotonia requiring brief NICU admission (< 24 hours) but was clinically classified as ChDM. Abbreviation: NICU, neonatal intensive care unit.

The most frequent perinatal complications in children with CDM were the presence of hypotonia at birth (93%), respiratory distress (75%), and feeding issues (91%). These complications led to a stay in the NICU in 91% of children with CDM. Admission lasted longer than 48 hours in 83% of children. Fifty‐two per cent of patients with CDM required intubation during NICU admission. Conversely, only one patient with a CTG trinucleotide repeat expansion of 400, still classified as E1 (200–500 CTG trinucleotide repeats), had mild neonatal hypotonia requiring a brief admission to the NICU but was still clinically classified as ChDM because of complete recovery of the clinical picture within the first 48 hours of life and the onset of cognitive issues noted at school age. None of the patients with ChDM had respiratory distress or feeding issues (Figure 1).

**FIGURE 2:**
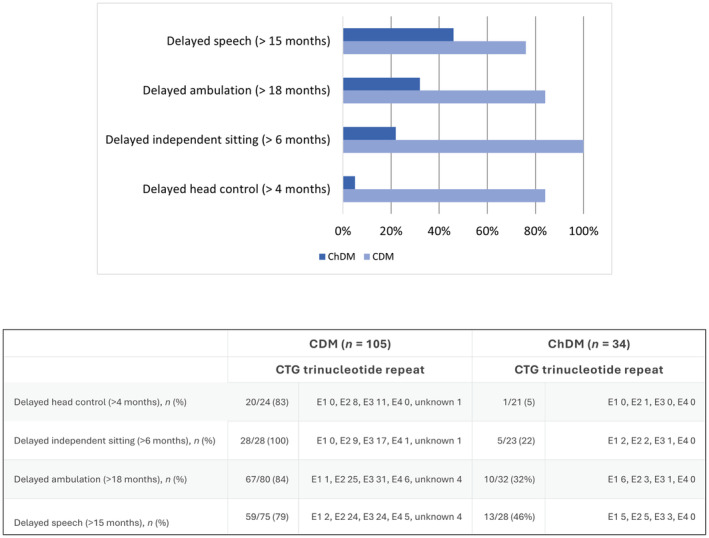
Delayed developmental milestones in children with congenital myotonic dystrophy (CDM) and childhood‐onset myotonic dystrophy (ChDM). The classes of CTG expansion are: E1 (200–500 CTG trinucleotide repeats); E2 (500–1000 CTG trinucleotide repeats); E3 (1000–1500 CTG trinucleotide repeats); and E4 (> 1500 CTG trinucleotide repeats).^14^

Developmental milestones were acquired with significant delay in most children with CDM. Eighty‐four per cent acquired independent ambulation with delay (after 18 months of age); 79% had speech delay (first meaningful words uttered after 15 months of age). Ambulation and speech delay were identified in 32% and 46% of patients with ChDM respectively (Figure 2).

Eighty‐three per cent of children with CDM and 36% of children with ChDM had an IQ below 70. Most children with CDM and extremely low cognitive function had a CTG trinucleotide repeat expansion classified as either E2 (500–1000 CTG trinucleotide repeats) or E3 (1000–1500 CTG trinucleotide repeats). Conversely, most children with ChDM with an IQ below 70 had a CTG trinucleotide repeat expansion classified as E1 (200–500 CTG trinucleotide repeats).

**FIGURE 3:**
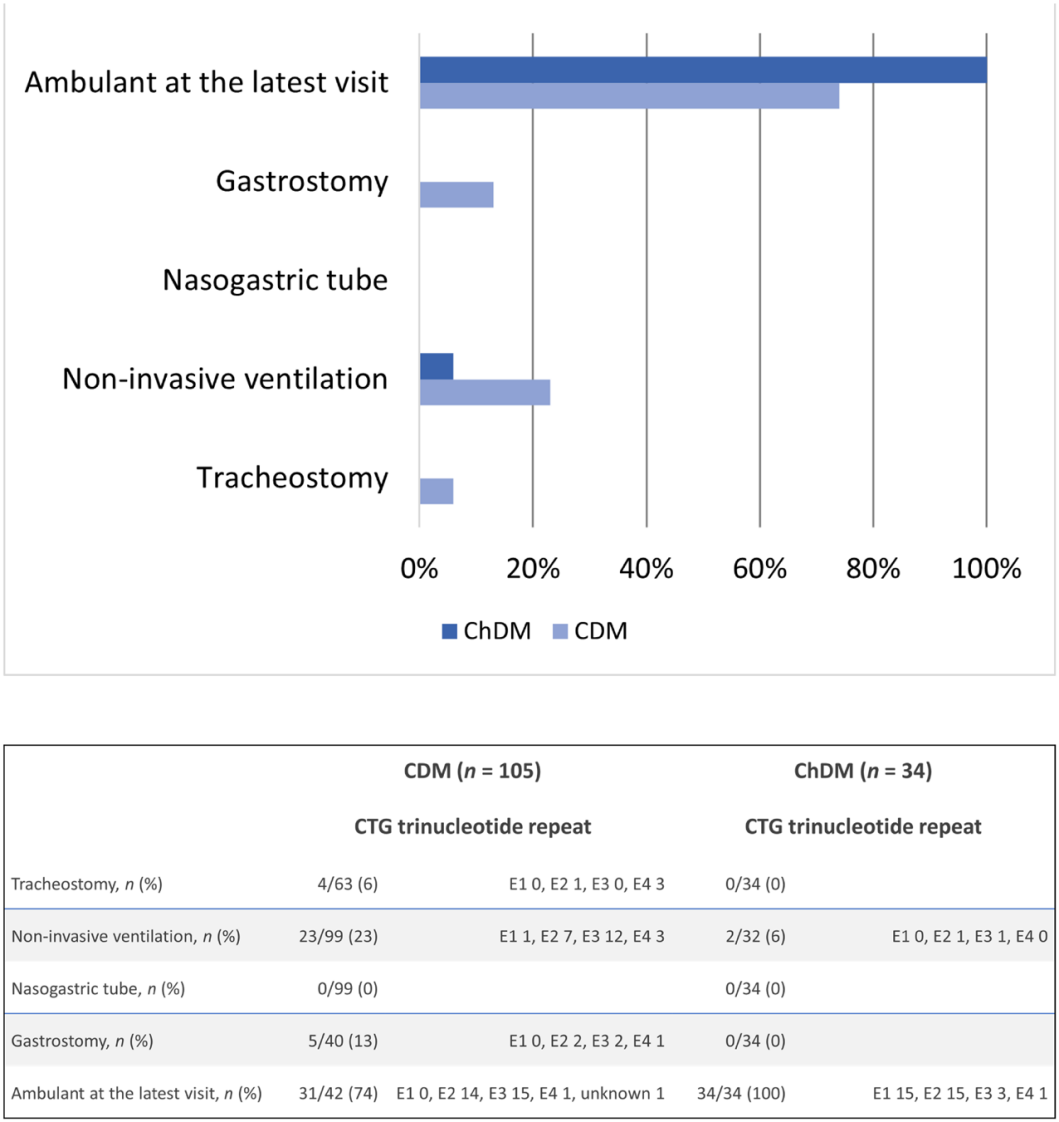
Respiratory and nutritional status at the patient's latest visit. At the latest available visit, children with congenital myotonic dystrophy (CDM) had a median age of 8 years 8 months (IQR = 5 years 2 months to 13 years 2 months) and children with childhood‐onset myotonic dystrophy (ChDM) had a median age of 12 years 2 months (IQR = 7 years 8 months to 16 years 4 months). The classes of CTG expansion are: E1 (200–500 CTG trinucleotide repeats); E2 (500–1000 CTG trinucleotide repeats); E3 (1000–1500 CTG trinucleotide repeats); and E4 (> 1500 CTG trinucleotide repeats).^14^

At the latest available visit, children with CDM had a median (IQR) age of 8 years 8 months (5 years 2 months to 13 years 2 months) while children with ChDM had a median (IQR) age of 12 years 2 months (7 years 8 months to 16 years 4 months). Seventy‐four per cent of children with CDM and all 34 children with ChDM were ambulant at the latest visit. Respiratory support was only required in children with CDM. Twenty‐three per cent used nocturnal non‐invasive ventilation and 6% still had a tracheostomy at their latest visit. Six per cent of children with ChDM required non‐invasive ventilatory support and none had a tracheostomy. Thirteen per cent of children with CDM and none of the children with ChDM had a gastrostomy at the latest assessment (Figure 3).

## DISCUSSION

Diagnostic delay in DM1 is a well‐known feature of the disease.^13^ However, data on how such diagnostic delay affects family planning are limited. Previous studies in relatively small groups^17^, ^18^ showed that the percentage of females with DM1 who were diagnosed because of a child affected by CDM ranged from 39% to 100%. The present work provides additional evidence from a large international population of 139 couples who had children affected by paediatric‐onset DM1. Only in half of these couples (69 of 139), the affected individual was aware of the diagnosis before their offspring was conceived. In 103 of 105 children born with congenital DM1, the disease was inherited from the mother. In almost half of these females (50 of 103; 48%) the diagnosis of DM1 was made only when the prenatal or postnatal characteristics of their offspring fulfilled the criteria for CDM. Even within the subgroup of patients with childhood‐onset disease, less than half of parents were aware of their diagnosis before family planning. A delay in diagnosis can be explained by the lack of awareness patients have of the disease and factors related to its multiple organ presentation, which may complicate the clinical scenario for non‐neuromuscular specialists. It can often be difficult to connect extramuscular symptoms to the underlying myotonic muscle disorder. Additionally, patients often underestimate myotonia when it appears as the first sign of the disease, as reported previously.^11^

Knowing the parental diagnosis is key to implementing genetic counselling, informing families on the risk in an affected offspring, and planning pregnancy from preconception to delivery, given the associated risk for affected females^17^, ^18^, ^19^ and children.^15^

Current care recommendations suggest offering genetic counselling to all affected individuals of childbearing age to enable them to make an informed decision about whether to proceed to genetic testing. Families should also be informed about the high risk of having a second child affected with the most severe form of DM1, having had a first child with CDM. Pre‐implantation genetic diagnosis is suggested by the most recent recommendations (https://www.myotonic.org/sites/default/files/pages/files/MDF_Pregnancy_Guideline.pdf), although no data on its outcome are currently available.^15^

Although this study was retrospective and did not focus on the reasons for such a diagnostic delay, it is worth noting that only 17% of the couples with a known diagnosis of DM1 decided to screen for DM1 prenatally. The decision to opt for a prenatal diagnosis was less likely when the affected individual was the mother. Indeed, only 7% of couples giving birth to children with CDM decided to conduct prenatal testing. Considering the high level of experience of the centres involved in this study, it is unlikely that the inheritance pattern and potential outcomes had not been discussed between referring physicians and either mothers or fathers. However, in other developmental conditions, families had mixed preferences and motivations for acquiring prognostic genetic information about their children.^20^, ^21^ Other reasons may have brought parents to avoid prenatal screening, such as personal beliefs and religious or ethical considerations. On the other hand, it is possible that some families opted not to test even after counselling as it would have made no difference to their decision‐making. These families may have decided to continue the pregnancy despite the associated difficulties.

The results of this study further strengthen findings suggesting a lack of correlation between the severity of symptoms and CTG trinucleotide repeat expansion in paediatric‐onset DM1, underlining that the extent of CTG trinucleotide repeat expansion cannot be taken as a predictor of disease severity, making prenatal counselling even more challenging.^22^

Detailed additional information on the complications associated not only with congenital, but also with childhood‐onset DM1 have been provided in this study. The most frequent prenatal complications associated with congenital DM1 were polyhydramnios, reduced fetal movements, and ventriculomegaly, with a prevalence of 51%, 51%, and 26% respectively, while none of the patients with ChDM had prenatal complications. These results are in line with previous studies.^23^, ^24^, ^25^, ^26^

This study supports that the disease burden in DM1 is high for children, families, and health care professionals; this applies not only at birth, but throughout disease progression, despite the motor, respiratory, and nutritional prognoses being considered benign from birth to childhood in CDM.^22^, ^27^, ^28^ More than half of the children with CDM in this group (52%) required intubation as early as the first day of life, as in a recent report by Omura et al.;^29^ 91% required admission to the NICU. Feeding issues requiring a nasogastric tube were reported in 82% of children at birth. Data at the latest available visit confirmed a trend towards reduced intensity of care, particularly regarding respiratory and nutritional support. At a median age of almost 9 years, only 6% children with CDM had tracheostomy and 23% required non‐invasive ventilation support. Thirteen per cent of children had a gastrostomy placed to supplement nutrition because of swallowing issues. Notably, delayed milestones and extremely low cognitive function were quite high, with over 80% of children with CDM being diagnosed with an IQ below 70.

In children with ChDM, musculoskeletal impairment was mild;^26^ although less severe than in CDM, cognitive and behavioural difficulties were reported. Only one patient (3% of the total ChDM sample) had mild hypotonia at birth, which improved in the first hours after birth; no patients had respiratory distress requiring ventilatory support. Ventilatory and nutritional support did not change significantly over time, with patients being clinically stable at a median age of 12 years. Only two patients (6% of the ChDM sample), required nocturnal non‐invasive ventilation because of sleep‐disordered breathing; none needed either a gastrostomy or nasogastric tube at the latest visit. Conversely, low cognitive function and delayed speech were reported in more than 30% of children with ChDM.

In line with previous studies,^22^, ^27^ this work highlights that, particularly in children with CDM, a multidisciplinary and highly competent approach is required to manage the expanding burden of the disease over subsequent generations. Current care recommendations suggest that for mothers known or suspected of carrying a child with DM1, a range of strategies aimed at limiting complications should be put in place. The delivery should be planned to benefit from multidisciplinary, highly specialized support, such as a high‐risk obstetrician and neonatal specialist. At birth, children with CDM will often need management in the NICU, which can deal with breathing and feeding support.^15^

The data from this large international population confirm and strengthen previous cross‐sectional studies on the outcomes and burden of disease in children with myotonic dystrophy. The collaborative international network of tertiary care neuromuscular centres looking after families with DM1 had a pivotal role in the development of this study. Data gathered were collected regularly by centres as part of a joint effort aimed at defining the natural history of paediatric DM1. However, given the retrospective nature of the study, clinical data on patient outcomes, such as the requirement for a nasogastric tube or non‐invasive ventilation, were not retrieved. Also, information regarding the decision of families towards a prenatal diagnosis, or how family planning was considered in detail before pregnancy, was missing. Lack of information regarding stillbirth or children who did not survive immediately after birth limited the possibility of establishing the exact prevalence of families who had a diagnosis because of the affected offspring. The question whether a more or less positive outcome at birth in affected children was associated with a known parental diagnosis was difficult to address in the present study because the clear definition of a causative association was limited by several factors. First, most information was gathered from medical charts. Second, the interrogation of data refers to a long retrospective time span and neonatal support techniques may have improved over time.

In conclusion, the results of this study highlight that an early diagnosis and proactive management in paediatric DM1 is paramount to treat complications; family planning may be affected by a delay in parental diagnosis. Parental decision to conduct prenatal testing despite the known clinical outcomes was also a significant finding of the study.

## CONFLICT OF INTEREST STATEMENT

The authors have stated that they had no interests which might be perceived as posing a conflict or bias.

## Data Availability

The data that support the findings of this study are available from the corresponding author upon reasonable request.
